# Acute Treatment of Ischemic Stroke Caused by Stent Graft Thrombosis After Thoracic Endovascular Aortic Repair

**DOI:** 10.7759/cureus.64465

**Published:** 2024-07-13

**Authors:** Takashi Saito, Takuya Saito, Tatsuhito Ishii, Kazunari Homma, Yoshifumi Kunii, Masaaki Koide, Toshihiko Ohashi

**Affiliations:** 1 Department of Neurology, Seirei Hamamatsu General Hospital, Hamamatsu City, JPN; 2 Department of Cardiovascular Surgery, Seirei Hamamatsu General Hospital, Hamamatsu City, JPN

**Keywords:** artery bypass, ischemic stroke treatment after tevar, endovascular aneurysm repair, aortic dissection, carotid arteries, thrombectomy

## Abstract

Planning for the acute phase of ischemic stroke in postoperative patients with aortic dissection is difficult from the perspective of concerns about worsening disease related to aortic dissection due to intravenous thrombolytic agents and securing access routes when mechanical thrombectomy is planned. Herein, we report that a 52-year-old man underwent thoracic endovascular aortic repair for acute type B aortic dissection. One year after the procedure, the patient developed a stroke caused by stent graft thrombosis, and computed tomography angiography showed occlusion of the left common carotid artery and left internal carotid artery. Stroke neurologists performed mechanical thrombectomy via a direct approach from the left common carotid artery, and successful recanalization was achieved. Furthermore, ligation of the proximal portion of the left common carotid artery and bypass surgery on the distal portion of the left common carotid artery were performed by cardiovascular surgeons. Although the patient had a postoperative hemorrhagic infarction, he returned to work without a recurrence of stroke after two years of follow-up. A direct carotid artery puncture we performed is an alternative in cases of anatomical difficulty or an unfavorable aortic arch. This case highlights not only the significance of interdisciplinary collaboration between cardiac and neurological specialists but also the impact of training dual-specialty cerebrovascular neurosurgeons on patient outcomes.

## Introduction

Thoracic endovascular aortic repair (TEVAR) is a less invasive and safer treatment than open surgery for aortic pathologies [1] and is widely performed as a standard treatment [2]. Mechanical thrombectomy (MT) is widely and commonly performed for acute large-vessel occlusion (LVO). Therefore, the access route for MT and secondary prevention of ischemic stroke should be considered in patients who have undergone TEVAR. Herein, we report a case of acute LVO caused by stent graft thrombosis post-TEVAR. A direct carotid artery puncture we performed is an alternative in cases of anatomical difficulty or an unfavorable aortic arch. MT and secondary prevention treatments were administered in a single phase, and the patient subsequently had a favorable outcome due to the minimally invasive approach that was achieved. 

## Case presentation

**Figure 1:**
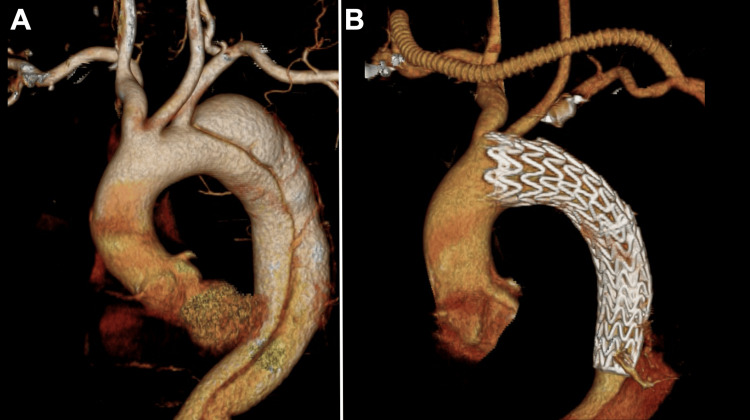
Thoracic endovascular aortic repair for type B aortic dissection. Computed tomography angiography shows a type B aortic dissection and a thoracic descending aortic dissecting aneurysm (A). Thoracic endovascular aortic repair with a right-to-left axillary artery bypass was performed (B).

A 52-year-old man with no medical history was diagnosed with type B aortic dissection and a descending dissecting thoracic aortic aneurysm. Computed tomography angiography (CTA) revealed a tear in the descending aorta distal to the origin of the left subclavian artery (LSA); the dissection extended to the abdominal aorta (Figure 1 A). TEVAR with a right axillary-to-left axillary artery bypass was performed. The stent graft partially overlapped the origin of the left common carotid artery (CCA); however, left CCA reconstruction was not performed because of good blood flow (Figure 1 B).

**Figure 2:**
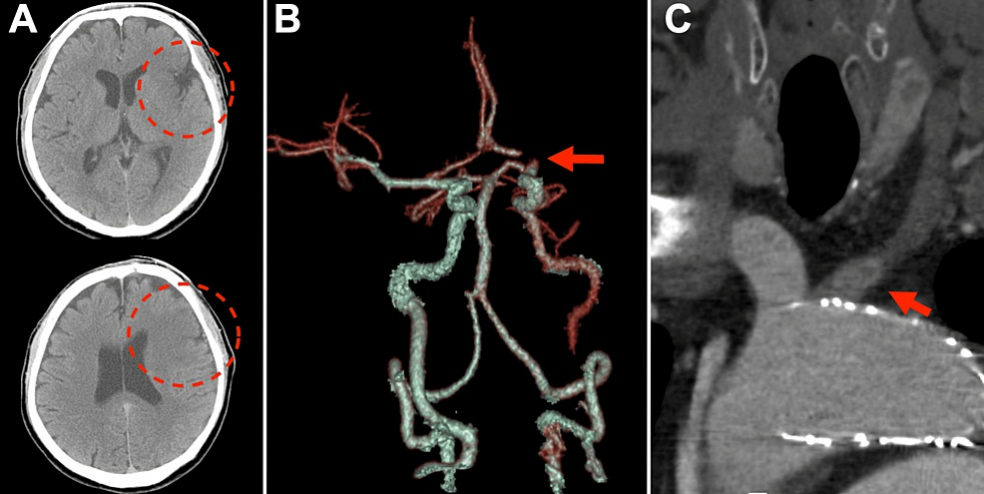
Computed tomography and computed tomography angiography on admission. Computed tomography shows an acute ischemic stroke in the left middle cerebral artery area. The upper one is at the level of the basal ganglia, and the lower one is at the level of the lateral ventricles (A). Computed tomography angiography (CTA) shows tandem occlusion of the proximal left common carotid artery (CCA) and terminal portion of the left internal carotid artery (B). The arrow indicates the occlusion of the proximal left CCA in the coronal section (C).

The patient continued with two antiplatelet therapies. One year later, he experienced difficulties in communication and physical movements. He was transferred to our hospital 10 hours after being last known. He presented with aphasia, right hemiparesis, and right hemianesthesia (National Institutes of Health Stroke Scale score = 11). Computed tomography (CT) showed an ischemic stroke of the left middle cerebral artery (MCA) (Alberta Stroke Program Early CT Score = 5). The CTA showed tandem occlusion of the proximal left CCA and the terminal portion of the left internal carotid artery (ICA) (Figure 2). 

**Figure 3:**
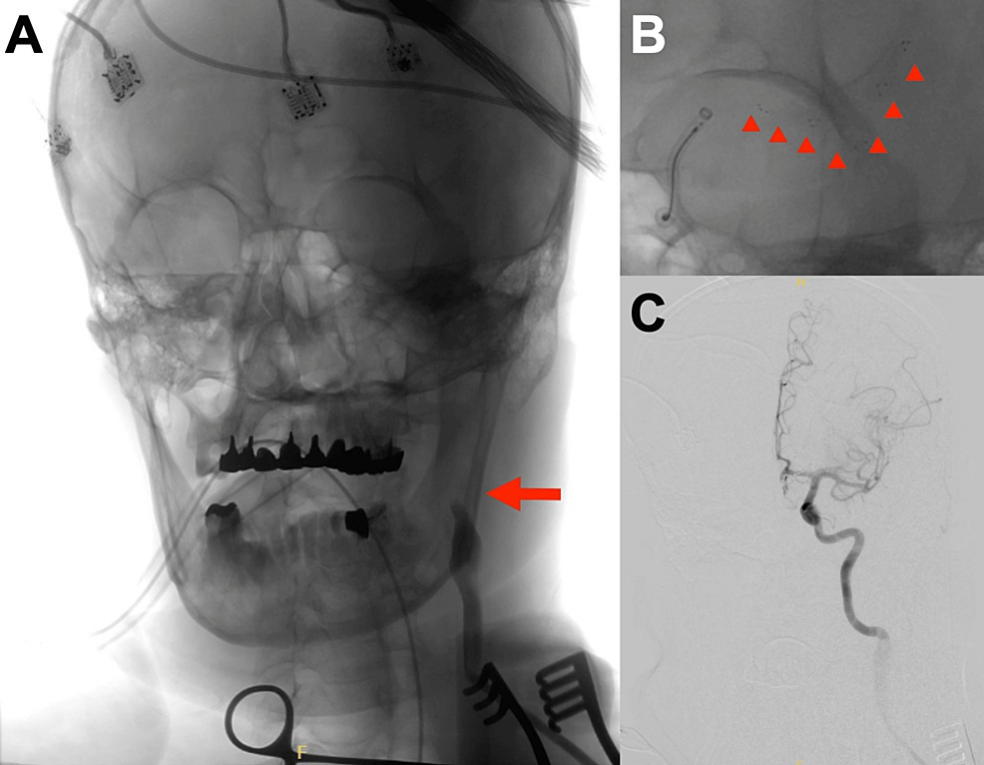
Angiography of the mechanical thrombectomy. Digital angiography of the sheath shows the left internal carotid artery (ICA) occlusion (A). A Solitaire X 4 × 40 mm is deployed from the left middle cerebral artery (MCA) M2 segment to the terminal portion of the left ICA. REACT71 is navigated to the proximal part of the left MCA M1 segment (B). Digital subtraction angiography after one pass shows successful recanalization (C).

He was diagnosed with left proximal CCA occlusion caused by stent graft thrombosis and occlusion of the terminal portion of the left ICA resulting from an artery-to-artery embolism. Accessing the left ICA via the aortic arch was difficult because of the overlapping stent graft origin of the CCA. Proximal CCA catheterization was also considered a risk factor for new thromboembolisms. Accordingly, MT was attempted via a direct puncture of the left CCA. Cardiovascular surgeons made a small skin incision in the left neck with the patient in the right neck rotation position under general anesthesia in a hybrid operating room. The left CCA was punctured with an 18-gauge needle, and a 9-foot-short sheath was placed. A 9-Fr OPTIMO balloon-guided catheter (Tokai Medical Products Inc., Kasugai, Aichi, Japan), REACT71 (Medtronic, Minneapolis, MN, USA), Phenom microcatheter (Medtronic), and CHIKAI14 microwire (Asahi Intec Co., Ltd., Seto, Aichi, Japan) were used by neurologists to access the terminal portion of the left ICA occlusion. A Solitaire X (4 × 40 mm [Medtronic, Minneapolis, MN, USA]) was deployed from the left MCA M2 segment to the terminal portion of the left ICA; REACT71 was navigated to the proximal part of the left MCA M1 segment. REACT71 was aspirated using an aspiration pump; REACT71 and Solitaire X were retrieved. Successful recanalization of thrombolysis in cerebral infarction 2b was achieved (Figure 3). 

**Figure 4:**
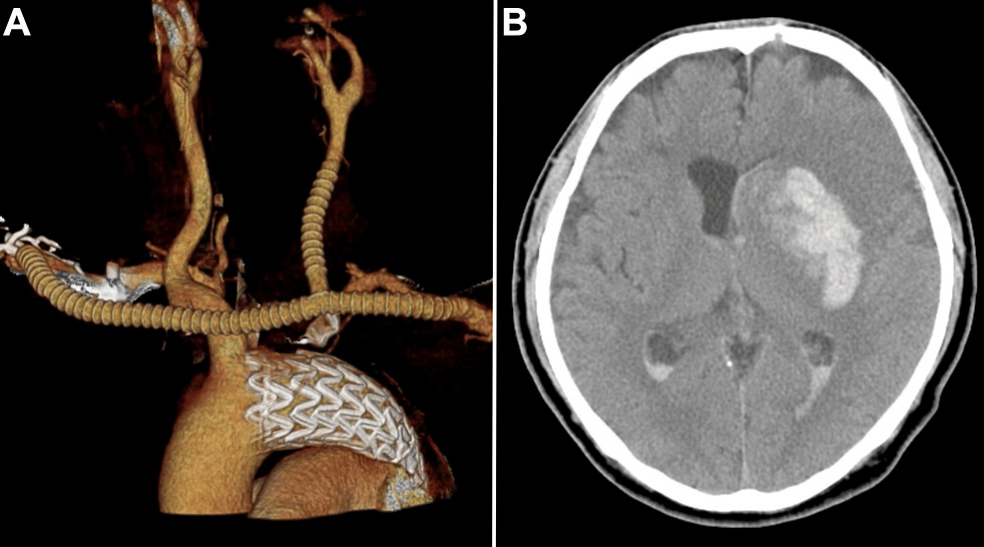
Postoperative computed tomography and computed tomography angiography. Ligation of the proximal part of the left common carotid artery (CCA) and bypass from the right axillary to the left axillary artery to the distal part of the left CCA (A). Postoperative computed tomography showed hemorrhage in the left putamen and caudate nucleus (B).

Subsequently, cardiovascular surgeons ligated the proximal part of the left CCA and performed an end-to-end graft to the CCA and an end-to-side graft to the graft anastomosis from the right axillary to the left axillary artery bypass graft to the distal part of the left CCA using a propaten vascular graft (WL Gore, Flagstaff, AZ, USA). Postoperative CT revealed a hemorrhage in the left putamen and caudate nucleus (Figure 4). 

The patient was managed with sedative hypotension for three days. Aphasia and right hemiparesis improved; the patient was moved to another hospital for rehabilitation. He could walk and return home with a modified Rankin scale score of 1 after three months. Returned to work without a recurrence of stroke after two years of follow-up.

## Discussion

The incidence of ischemic stroke in the perioperative period of TEVAR is 4.1% [3]. Stroke risk increases if the LSA is covered with a stent graft [4]. However, few studies have examined the long-term risks of ischemic stroke post-TEVAR. The long-term stroke risk may be high if the stent graft covers the LSA and overlaps with the carotid artery.

MT is commonly performed via a transfemoral or transbrachial approach. A direct carotid artery puncture is an alternative in cases of anatomical difficulty or an unfavorable aortic arch [5]. Direct carotid artery puncture may result in serious complications, such as dissection, hematoma formation, and carotid artery rupture [6]. However, direct carotid puncture with adequate CCA exposure can reduce these complications [7]. Here, the exposure of the carotid artery led to a safe procedure useful for subsequent ligation and bypass surgery.

Hemorrhagic transformation after an ischemic stroke is caused by blood-brain barrier disruption and reperfusion injury [8]. In addition, cerebral hyperperfusion syndrome (CHS) or vascular damage may occur after revascularization procedures such as MT or bypass surgery [9]. Both occurred immediately after revascularization. In this patient, the cause of the hemorrhagic transformation is unknown. However, identifying the risk of CHS or vascular damage before revascularization may help manage hemorrhagic transformation promptly.

The heart-brain team approach by heart and brain specialists is effective in treating patent foramen ovale [10] and acute myocardial infarction after acute stroke [11]. Because the heart and brain are highly specialized organs, specialists in either organ alone may not be capable of developing an optimal treatment plan. Here, cardiovascular surgeons and stroke neurologists discussed and formulated an acute treatment plan from a long-term perspective. The need for not only a heart-brain team approach but also training dural specialties cerebrovascular neurosurgeons is growing as the heart and brain fields are becoming more sophisticated in their treatment. This case study underscores not only the significance of interdisciplinary collaboration between cardiac and neurological specialists but also for training dual-specialty cerebrovascular neurosurgeons and highlights the potential influence of optimal access routes and advanced risk estimation on patient outcomes, considering the individual patient’s background factors.

## Conclusions

We report a case in which MT and surgery for the embolic source were performed at once for an ischemic stroke after TEVAR. Cardiovascular surgeons and stroke neurologists collaborated to perform MT via direct puncture of the left CCA, ligation of the left CCA, and bypass surgery of the distal portion of the left CCA. The exposure of the carotid artery led to a safe procedure useful for subsequent ligation and bypass surgery. This case highlights not only the significance of interdisciplinary collaboration between cardiac and neurological specialists but also the impact of training dual-specialty cerebrovascular neurosurgeons on patient outcomes.
